# Pharmacokinetics of Enrofloxacin in Plasma, Urine, and Feces of Donkey (*Equus asinus*) after a Single Intragastric Administration

**DOI:** 10.3390/antibiotics13040355

**Published:** 2024-04-12

**Authors:** Bowen Yang, Shijie Liu, Jie Cheng, Honglei Qu, Yanxin Guo, Chuanliang Ji, Yantao Wang, Shancang Zhao, Shimeng Huang, Lihong Zhao, Qiugang Ma

**Affiliations:** 1 State Key Laboratory of Animal Nutrition, College of Animal Science and Technology, China Agricultural University, Beijing 100193, China; yangbw95@gmail.com (B.Y.); qintong@caas.cn (S.L.); quhl@dongeejiao.com (H.Q.); gyx18911847192@outlook.com (Y.G.); shimengh@cau.edu.cn (S.H.); zhaolihongcau@cau.edu.cn (L.Z.); 2 National Engineering Research Center for Gelatin-Based Traditional Chinese Medicine, Dong-E-E-Jiao Co., Ltd., Liaocheng 252201, China; chengjie@dongeejiao.com (J.C.); jicl@dongeejiao.com (C.J.); wangyt@dongeejiao.com (Y.W.); 3 Shandong Academy of Agricultural Sciences, Jinan 250100, China; shancangzhao@126.com

**Keywords:** enrofloxacin, ciprofloxacin, pharmacokinetics, donkey, urinary excretion

## Abstract

Enrofloxacin is a broad-spectrum antimicrobial agent, but the study of its pharmacokinetics/pharmacodynamics (PKs/PDs) in donkeys is rarely reported. The present study aimed to investigate the pharmacokinetics of enrofloxacin administered intragastrically, and to study the pharmacokinetics of enrofloxacin and its metabolite ciprofloxacin in plasma, urine, and feces, and the PK/PD parameters were investigated to provide a rationale for enrofloxacin treatment in donkeys. A total of five healthy donkeys were selected for intragastric administration of 7.5 mg·kg^−1^ BW of enrofloxacin by gavage, and blood, urine, and fecal samples were collected. The results showed that the elimination half-life of plasma enrofloxacin was 11.40 ± 6.40 h, T_max_ was 0.55 ± 0.12 h, C_max_ was 2.46 ± 0.14 mg·L^−1^, AUC_0–∞_ was 10.30 ± 3.37 mg·L^−1^·h, and mean residence time (MRT) was 7.88 ± 1.26 h. The T_max_ of plasma ciprofloxacin was 0.52 ± 0.08 h, C_max_ was 0.14 ± 0.03 mg·L^−1^, and AUC_0–∞_ was 0.24 ± 0.16 mg·L^−1^·h. Urinary C_max_ was 38.18 ± 8.56 mg·L^−1^ for enrofloxacin and 15.94 ± 4.15 mg·L^−1^ for ciprofloxacin. The total enrofloxacin and ciprofloxacin recovered amount in urine was 7.09 ± 2.55% of the dose for 144 h after dosing. The total enrofloxacin and ciprofloxacin recovered amount in feces was 25.73 ± 10.34% of the dose for 144 h after dosing. PK/PD parameters were also examined in this study, based on published MICs. In conclusion, 7.5 mg/kg BW of enrofloxacin administered intragastrically to donkeys was rapidly absorbed, widely distributed, and slowly eliminated in their bodies, and was predicted to be effective against bacteria with MICs < 0.25 mg·L^−1^.

## 1. Introduction

Donkeys have a stoic character and adapt to complex environments, so they are often used as draft animals in underdeveloped areas [1]. In Western countries, donkeys are regarded as companion animals and pets [2]. In China, donkey meat is regarded as a delicacy, and donkey skin glue (*Asini Corii Colla*, Ejiao) is an extremely valuable and sought-after traditional Chinese medicine [3]. Donkeys are exposed to complex and austere environments when used as draft and production animals, resulting in the frequent occurrence of diseases. Many donkeys do not receive the same level of routine health care as horses, so they are often exposed to pathogenic bacteria such as *Streptococcus equi*, *Streptococcus zooepidemicus*, *Mycobacterium* spp., and *Burkholderia mallei* [4]. However, research on donkeys, especially in pharmacology, is still lacking. Drugs used in donkeys are rarely dosed based on evidence and in most cases are extrapolated from equines, which can lead to risks that compromise efficacy and side effects [5]. Compared to horses, donkeys appear to require higher doses of enrofloxacin to achieve adequate plasma concentrations [6]. Donkeys also appear to have a greater ability to metabolize or eliminate certain drugs than horses, possibly because they possess a higher number or activity of cytochrome P450 isoenzymes [7,8,9]. Improper use of veterinary clinical drugs, especially antibiotics, not only affects the health and production of animals but also has adverse effects on humans who consume these animal products, such as increased antibiotic resistance [10]. There is a lack of pharmacokinetic studies, resulting in problems with drug residues in edible tissues and milk and an inability to reasonably propose a withdrawal period based on drug metabolism. Therefore, it is crucial to study the specific pharmacokinetics of the drug in donkeys.

**Figure 1:**
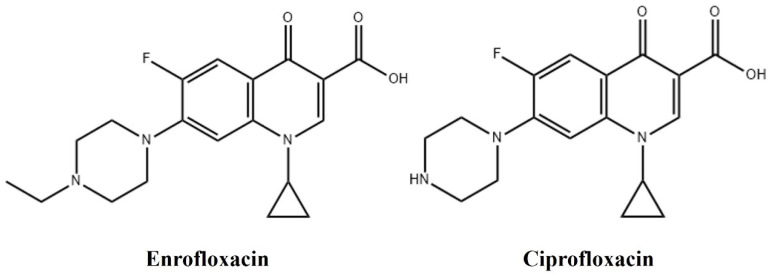
Chemical structural formulae of enrofloxacin and ciprofloxacin.

Enrofloxacin is a chemically synthesized fluoroquinolone antibiotic, also known as ethyl ciprofloxacin. Enrofloxacin binds to bacterial DNA gyrase subunit A, thus inhibiting the cleavage and ligation functions of the enzyme, preventing the replication of bacterial DNA, and presenting an antibacterial effect [11]. It is metabolized in the body to remove the ethyl group to produce ciprofloxacin, which still has strong antimicrobial activity [12]. The chemical structural formulae of enrofloxacin and ciprofloxacin are shown in Figure 1. Enrofloxacin has bactericidal activity against many Gram-negative aerobic bacteria (e.g., *Actinobacillus*, *Pasteurella* spp., and *Salmonella* spp.), and is moderately active against several Gram-positive aerobic bacteria (e.g., *Staphylococcus*, *Erythromyces equi*, and *Mycobacterium* spp.) [13]. Enrofloxacin is rapidly and completely absorbed orally [14]. After administration, concentrations in tissue are higher than that in plasma, which facilitates the treatment of systemic and deep-tissue infections [15]. Approximately 15–50% of enrofloxacin is eliminated and excreted from the body in its parent compound [16]. In the US, enrofloxacin is approved for the treatment of acute respiratory diseases caused by bacterial infections in edible animals [17]. It has also been shown to treat infectious diseases in sheep caused by sensitive pathogens, including *Corynebacterium pseudotuberculosis*, *Staphylococcus aureus*, *Streptococcus agalactiae, S. dysgalactiae*, *Mycoplasma agalactiae*, *Escherichia coli*, and *Haemophilus somnus* [18]. In the UK, enrofloxacin is a commonly used antibiotic in equine practice [19]. Given these pieces of evidence, enrofloxacin is a highly promising antibacterial agent for use as a pathogen infection countermeasure for donkeys. However, the pharmacokinetics of enrofloxacin and its metabolite ciprofloxacin when administered intragastrically in donkeys have not been reported. Therefore, the present study investigated the pharmacokinetics of a single intragastric administration of enrofloxacin in plasma, urine, and feces and analyzed the pharmacodynamics based on published MICs of the pathogenic bacteria with a view to provide prerequisites for the rational use of enrofloxacin in donkeys.

## 2. Results

### 2.1. HPLC Method Validation

The method used to determine the concentration of enrofloxacin and ciprofloxacin in plasma, urine, and feces was validated. The accuracy of enrofloxacin ranged from 83.49% to 116.88%, and that of ciprofloxacin ranged from 91.03% to 114.42%. The LOQs were 0.01 μg mL^−1^ for enrofloxacin and 0.02 μg mL^−1^ for ciprofloxacin. The signal-to-noise ratio (S/N) was ≥3 for LOD and ≥10 for LOQ. Linearity was observed in the range of 50 ng mL^−1^ to 2000 ng mL^−1^ and regression equations were calculated with correlation coefficients (R^2^) ≥ 0.99. The mean recoveries were ≥ 85% for both enrofloxacin and ciprofloxacin.

### 2.2. Pharmacokinetic Parameters of Enrofloxacin and Its Metabolite Ciprofloxacin in the Plasma of Donkeys

The plasma pharmacokinetic parameters of enrofloxacin and its metabolite ciprofloxacin in donkeys after a single intragastric administration of 7.5 mg·kg^−1^ BW are shown in Table 1. It can be seen that after gavage administration, the plasma concentration of enrofloxacin in donkeys reached a C_max_ of 2.46 ± 0.14 mg·L^−1^ at a T_max_ of 0.55 ± 0.12 h. The AUC_0–∞_ was 10.30 ± 3.37 mg·L^−1^·h. These results indicate that enrofloxacin is rapidly absorbed and slowly eliminated by the donkeys after administration.

The parameters for ciprofloxacin, a metabolite of enrofloxacin, are also listed in Table 1. The concentration of ciprofloxacin peaked at 0.14 ± 0.03 mg·L^−1^ at 0.52 ± 0.08 h after enrofloxacin administration. Ciprofloxacin exposure in donkeys appears to be extremely low relative to enrofloxacin exposure (plasma ciprofloxacin AUC is less than 2.5% of enrofloxacin AUC).

**Figure 2:**
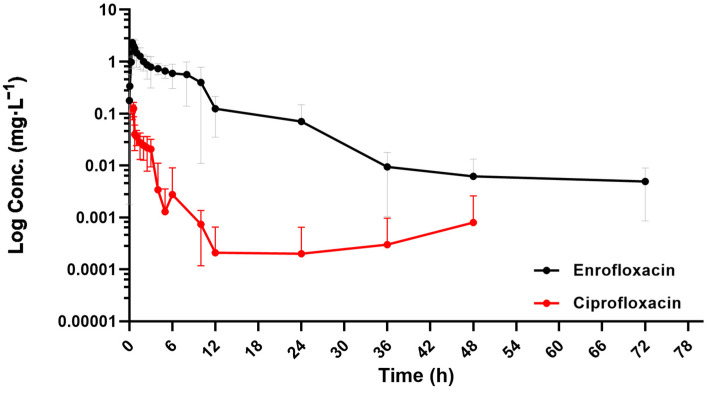
Concentration vs. time plots for plasma enrofloxacin and its metabolite ciprofloxacin in donkeys after a single intragastrical administration of 7.5 mg·kg^−1^ BW enrofloxacin, *n* = 5.

As is shown in Figure 2, enrofloxacin was first detected in plasma at 0.08 h after gavage and rapidly increased to a maximum concentration of 2.46 ± 0.14 mg·L^−1^ over time. After 36 h, enrofloxacin was barely detectable in plasma. As for ciprofloxacin, the first detectable time was 0.42 h, followed by a peak of 0.14 mg·L^−1^ at 0.52 h. The plasma concentrations of ciprofloxacin were low during the whole period and almost undetectable after 6 h.

### 2.3. Pharmacokinetic Parameters of Enrofloxacin and Its Metabolite Ciprofloxacin in the Urine of Donkeys

The urinary pharmacokinetic parameters after a single intragastric administration of 7.5 mg·kg^−1^ BW enrofloxacin and its metabolite ciprofloxacin in donkeys are presented in Table 2. The rate of urinary excretion of enrofloxacin reached a maximum of 4.36 ± 1.10 mg·h^−1^ at 19.75 ± 1.30 h after administration. These results were similar to the plasma results and the time taken for enrofloxacin to be excreted in donkeys was longer. As for ciprofloxacin, the rate of urinary excretion of enrofloxacin reached a maximum of 1.82 ± 0.98 mg·h^−1^ at 16.00 ± 3.00 h after administration.

The amounts of enrofloxacin and ciprofloxacin cumulatively recovered in urine were 46.85 ± 10.32 and 30.42 ± 6.54 mg. Enrofloxacin and ciprofloxacin combined were recovered in (7.09 ± 2.55)% of the total amount administered.

**Figure 3:**
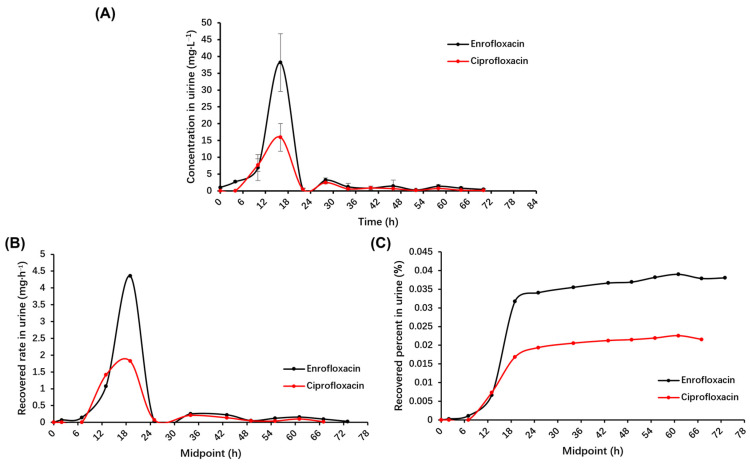
The excretion of enrofloxacin and its metabolite ciprofloxacin in the urine of donkeys after a single intragastric administration of 7.5 mg·kg^−1^ BW, *n* = 5: (**A**) Plots of mean concentrations of urine enrofloxacin and its metabolite ciprofloxacin vs. time in donkeys. (**B**) Plots of the recovered rate of enrofloxacin and its metabolite ciprofloxacin in urine vs. midpoint in donkeys. (**C**) Plots of the recovered percent of enrofloxacin and its metabolite ciprofloxacin in urine vs. midpoint in donkeys. Midpoint, the midpoint of the sampling start and end times.

As shown in Figure 3A, enrofloxacin was first detected in urine at 6 h, peaked at 18 h, and then its levels decreased rapidly. The trend for ciprofloxacin was similar to that of enrofloxacin. Both drugs were undetectable after 72 h.

It can be seen in the rate curves that the excretion rate peaks around 18 h (Figure 3B). The rate then decreases rapidly until it nearly goes to zero after 72 h. There was a corresponding trend in terms of the amount of recovery, with the incremental increase in the cumulative recovery amount slowing down from about 18 h and approaching a horizontal trend after 72 h (Figure 3C). The changing trends of enrofloxacin and ciprofloxacin are similar.

### 2.4. Pharmacokinetic Parameters of Enrofloxacin and Its Metabolite Ciprofloxacin in Feces of Donkeys

The cumulative amount of enrofloxacin recovered in the feces after a single intragastric administration of 7.5 mg·kg^−1^ BW enrofloxacin in donkeys was 261.06 ± 49.22 mg and the recovered amount of ciprofloxacin was 0.33 ± 0.07 mg; the total of the two drugs accounted for (25.73 ± 10.34)% of the total amount administered. Combined with the urine results, it is known that enrofloxacin is absorbed completely and excreted in low amounts.

**Figure 4:**
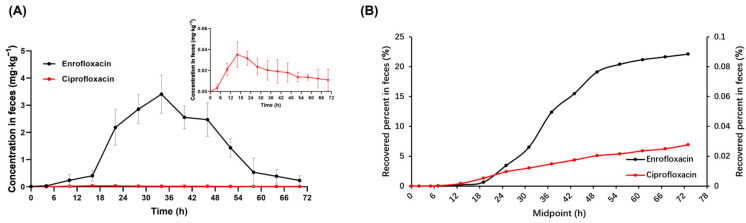
The excretion of enrofloxacin and its metabolite ciprofloxacin in the feces of donkeys after a single intragastric administration of 7.5 mg·kg^−1^ BW, *n* = 5: (**A**) Plots of mean concentrations in feces of enrofloxacin and its metabolite ciprofloxacin vs. time in donkeys. (**B**) Plots of the recovered percent of enrofloxacin and its metabolite ciprofloxacin in feces vs. midpoint in donkeys. Midpoint, the midpoint of the sampling start and end times.

The trend of fecal content of enrofloxacin and ciprofloxacin over time is shown in Figure 4A. Enrofloxacin was first detected in the feces at around 12 h and reached a maximum concentration of 34.09 mg·kg^−1^ at around 36 h. Enrofloxacin was undetectable in the feces after 108 h. As for ciprofloxacin, it was first detected in the feces at 6 h, reached a maximum concentration of 0.04 mg·kg^−1^ at around 18 h, and was undetectable after 90 h. The recovered amount of enrofloxacin and ciprofloxacin in the feces increased rapidly at first and then tended to flatten (Figure 4B).

### 2.5. PK/PD Parameters for Enrofloxacin and Ciprofloxacin in Donkeys

The pharmacodynamics of enrofloxacin were studied to determine the effective dose. We calculated and presented the AUC_24_/MIC for plasma (Table 3). The minimum inhibitory concentrations (MICs) of several bacteria isolated from equines are listed based on previous studies in Table 4 [20]. According to the two tables, the dose of enrofloxacin in our study was predicted to be effective for pathogenic bacteria with MICs of 0.03 (*Salmonella* spp., *E. coli*, *T. equigenitalis*, *Klebsiella* spp., and *A. equuli*), 0.06 (*Staphylococcus* spp.), and 0.12 (*Proteus* spp.) mg·L^−1^ (AUC_24_/MIC ≥ 50). However, enrofloxacin is not an effective option for *Strept. Zooepidemicus*, *P. aeruginosa*, *R. equui*, *Strept. equi*, or *Strept. equisimilis.*

## 3. Materials and Methods

### 3.1. Chemicals and Reagents

The enrofloxacin (enrofloxacin hydrochloride, 98%) was purchased from Zhongmu Nanjing Animal Pharmaceutical Co., Ltd. (Nanjing, China).

The concentrations of enrofloxacin and ciprofloxacin standard solutions in methanol were ≥99.0%, and those of enrofloxacin-d5 solution in methanol and ciprofloxacin-d8 hydrochloride solution in acetonitrile were ≥99.0%, and 95.0%. The standard solutions mentioned above were purchased from the Research and Monitoring Institute of Environmental Protection, Ministry of Agriculture, Tianjin, China. Hydrochloric acid and n-hexane were both analytically pure reagents, while formic acid, acetonitrile, methanol, and sodium hydroxide were chromatographically pure and used for HPLC in this study.

### 3.2. Animals

Animal experiments were conducted in accordance with protocols approved by the Institutional Animal Care and Use Committee (IACUC) of China Agricultural University (grant No. AW80803202-1-3). A total of 5 fattening male donkeys were selected with body weights of 171, 185, 158, 105, and 119 kg, with an average body weight of 147.60 ± 34.20 kg. The donkeys were obtained from Dong-E-E-Jiao Co., Ltd., Liaocheng, China. All animals were acclimatized for at least 7 days before the formal trial. Physical and biochemical examinations were performed to ensure that all the donkeys were healthy. At 2 h before the trial, each donkey was guided into a metabolic cage (2 m × 0.8 m × 1.8 m). No donkeys were fasted before administration. Each donkey was fed with 1.50 kg of concentrate feed and were free to drink water and ingest grain straw every day during the trial period of 7 days. Samples from plasma, urine, and feces were collected before the donkeys were administrated with enrofloxacin solution and were used as blank samples. See more information on the donkeys in Table 5. No adverse reactions were observed in the donkeys during the trial period. No adverse changes were observed in physical examination, blood tests, or blood biochemistry.

### 3.3. Experimental Design

The donkeys were intragastrically administrated a single dose of 7.5 mg/kg BW enrofloxacin by gavage. The dose administered was referenced from a previous study in equines [21]. Enrofloxacin was administered as a solution of enrofloxacin hydrochloride water. After administration, blood samples were collected from each donkey through the anterior vena cava and put into anticoagulation (heparin sodium) tubes. Urine and feces samples were collected in bags and weighed every 6 h. The blood sample time collection points were set at 0.00, 0.08, 0.25, 0.42, 0.58, 0.75, 1.0, 1.5, 2.0, 2.5, 3, 4, 6, 8, 10, 12, 24, 48, and 72 h after administration. All the samples were kept at −80 °C until further analysis. The pharmacokinetic experiment lasted for 7 d.

### 3.4. Determination of Enrofloxacin

The concentrations of enrofloxacin and its metabolite ciprofloxacin in the plasma, urine, and feces of donkeys were determined using a high-performance liquid chromatography–triple-quadrupole tandem mass spectrometer equipped with a fluorescence detector (AB SCIEX QTRAP 5500, Sciex, Framingham, MA, USA). Enrofloxacin and ciprofloxacin were determined with reference to previous reports [22,23]. In brief, 1 g of the plasma, urine, or feces samples was weighed separately and put into a 50 mL centrifuge tube, and 50 μL of mixed internal standard working solution at a concentration of 1 μg·mL^−1^ was added. For the mixed internal standard working solution (1 μg·mL^−1^), 100 μg·mL^−1^ of enrofloxacin-d_5_ and ciprofloxacin-d_8_ isotope internal standard stock solution 0.1 mL were measured into a 10 mL volumetric flask, and the volume was fixed with methanol. Fecal samples were weighed after all the feces collected during a sampling period had been thoroughly mixed. Then, 10 mL of acidified acetonitrile was added, vortexed, and mixed for 1 min, ultrasonicated for 20 min, and centrifuged at 8000× *g* for 5 min. The extracts were mixed in 50 mL centrifugal cuvettes. The residue in the tube was repeatedly extracted with 10 mL of acidified acetonitrile, and then the two extraction solutions were mixed. An amount of 10 mL of hexane was added to the extraction solution and shaken for 10 min. The solution was centrifuged at 15,000× *g* for 5 min and the hexane layer was discarded. The acetonitrile layer was blown to near dryness with a nitrogen purger at 50 °C and dissolved with 2 mL of aqueous methanol (1:9, *v*/*v*), which was then poured into a 0.22 μm microporous filter membrane to obtain the sample solution.

The chromatographic column was Hypersil^TM^ BDS C18 (250 mm × 4.6 mm I.D., 5 µm; Thermo Scientific, Waltham, MA, USA). The mobile phase was 18% acetonitrile and 82% phosphate buffer (0.05%; pH = 2.8 adjusted by triethylamine), and its flow rate was set at 1 mL·min^−1^. The excitation and emission wavelengths of the fluorescence detector were 280 and 450 nm, respectively.

### 3.5. HPLC Method Validation

The validated methods in this study referred to the Guidelines for Validation of Analytical Methods (9101), *Chinese Pharmacopoeia*, 2020 Edition. For sample testing, 5 QC samples (50 ng·mL^−1^ of control solutions) were run at the very beginning, after which a QC sample was inserted once between each batch of assayed samples. Standard working solutions of enrofloxacin and ciprofloxacin were diluted with aqueous methanol (1:9, *v*/*v*) to make control solutions at concentrations of 50, 100, 200, 500, 1000, and 2000 ng·mL^−1^. The control solutions were measured sequentially from low to high concentrations, standard curves were plotted based on the ratio of the peak area of the control solution to the corresponding concentration, and regression equations and correlation coefficients were calculated. Recovery, limits of detection (LODs), and limits of quantification (LOQs) in three blank matrices (plasma, urine, and feces) were also analyzed.

### 3.6. Pharmacokinetic and Pharmacodynamic Analysis

The concentrations of enrofloxacin and ciprofloxacin were calculated from the assay results. The data analysis was performed by the non-compartmental analysis using a combined linear trapezoidal rule approach using Certara Phoenix WinNonlin (Ver 8.1; Pharsight Corp., Raleigh, NC, USA). Pharmacokinetic parameters were referenced from previous reports [24,25]. The peak plasma concentrations (C_max_) of enrofloxacin and ciprofloxacin as well as the times to reach peak concentration (T_max_) for the study were calculated from the individual plasma concentration–time curves. The areas under the plasma concentration–time curves for AUC_0–∞_ studies were calculated by the method of trapezoids. The mean residence time (MRT), plasma clearance (Cl/F), and volume of distribution (Vz/F) were also calculated. For the urine, curves of urinary excretion rate, cumulative recovered amount, the percentage of excretion by urine, and the area under the rate curve (AURC_0–∞_) were calculated, referring to previous reports [26]. For the feces, the cumulative recovered amount of enrofloxacin and ciprofloxacin and the percentage of extraction by feces were calculated. Data were reported as the $\bar{x}$ ± *sd*.

For the PK/PD analysis, published MICs of pathogenic bacteria associated with equids [20] were listed. The AUC_24_/MIC and C_max_/MIC of plasma and urine were calculated to determine the appropriateness of the administered dose.

## 4. Discussion

Enrofloxacin is a proven-effective broad-spectrum antimicrobial agent for animals that belongs to fluoroquinolones. It is characterized by high antibacterial activity and low toxicity [27]. Enrofloxacin is strongly lipophilic and can pass through cell membranes by passive transport with high distribution coefficients and membrane permeability coefficients [28]. These characteristics allow for good oral absorption and rapid and widespread tissue distribution of enrofloxacin. Enrofloxacin is metabolized in the body by de-ethylation to produce ciprofloxacin, a potent antimicrobial agent already used in human medicine [29]. The antimicrobial therapeutic efficacy of enrofloxacin in a number of livestock animals has been recognized and approved by governments [17]. However, for such a promising antimicrobial agent, studies on its enteral administration in donkeys have not been reported so far. Therefore, our study on the pharmacokinetics and pharmacodynamics of enrofloxacin in donkeys will provide experimental evidence for the rational use of this promising antimicrobial agent.

The present results provide evidence for the rapid absorption of enrofloxacin in donkeys, the high plasma concentration, and the long half-life of the drug. The AUC of plasma enrofloxacin was reported to be 22.74 ± 9.99 and 16.50 ± 4.24 μg·h·mL^−1^ for donkeys injected intravenously and intramuscularly with enrofloxacin at 5 mg/kg BW, respectively, which was much higher than that of the present study (10.30 ± 3.37 mg·L^−1^·h) [6]. Despite the species being the same, the results varied considerably because the intragastric route of administration is more influenced by the gastrointestinal tract. Also, for enteral administration, a single oral dose of enrofloxacin (7.5 mg·kg^−1^ BW) in non-pregnant mares resulted in a plasma half-life of 8.00 ± 2.20 h, a T_max_ of 1.25 h, a C_max_ of 1.78 ± 1.43 μg·mL^−1^, and an AUC_0–∞_ of 9.20 ± 2.17 μg·mL^−1^·h [14]. Oral administration of 5 mg·kg^−1^ BW enrofloxacin to horses was reported to have a half-life in serum of 7.75 h, a T_max_ of 0.92 ± 0.59 h, a C_max_ of 1.85 ± 0.86 μg·mL^−1^, and an AUC_0–∞_ of 18.94 ± 14.41 μg·mL^−1^·h [30]. In foals, the plasma half-life was 7.75 h, the T_max_ was 2.20 ± 2.17 h, the C_max_ was 2.12 ± 0.51 μg·mL^−1^, and the AUC_0–∞_ was 58.47 ± 16.37 μg·mL^−1^·h after oral administration of 10 mg·kg^−1^ BW of enrofloxacin [31]. The above reports in the genus *Equus* differ somewhat from the results of the present study. This may be due to the fact that donkeys have a higher metabolic rate and cellular water content compared to horses, and therefore the drug is metabolized quickly and has a short half-life in donkeys [32]. In the present study, after intragastric administration of enrofloxacin to donkeys, the plasma MRT was found to be 7.88 ± 1.26 h, Cl/F was 0.81 ± 0.28 L·kg^−1^·h^−1^, and Vz/F was 12.88 ± 6.70 L·kg^−1^, similarly to previously reported results [30,33]. It can be seen that the ability to metabolize enrofloxacin varies from animal to animal according to the reports mentioned above, which reinforces the need for pharmacokinetic studies in specific animals. In general, enrofloxacin is rapidly absorbed in donkeys with a long half-life and slow elimination.

In addition to plasma, changes in the concentrations of enrofloxacin and ciprofloxacin in urine and feces were also of interest. In the present study, the total amount of urinary enrofloxacin recovered was 46.85 ± 10.32 mg, and that of ciprofloxacin was 30.42 ± 6.54 mg, with the total urinary drug as a percentage of the administered dose being (7.09 ± 2.55)%. It has been reported that horses given multiple gavages of enrofloxacin have urine concentrations of enrofloxacin that exceed those in serum [21]. In the present study, the C_max_ of plasma was 2.46 ± 0.14 mg·L^−1^ and that of urine was 38.18 ± 8.56 mg·L^−1^. The total urinary excretion of enrofloxacin following intravenous administration of 5 mg·kg^−1^ BW of enrofloxacin to horses was reported to be 80.9 ± 23.4 mg, which represents (3.4 ± 0.9)% of the total amount of enrofloxacin administered [34]. Testing urine drug concentrations can help provide evidence for the treatment of kidney or urinary tract infections.

Enteral administration, both oral and intragastric, has the major disadvantage of being constrained by the gastrointestinal tract. Some drugs may be excreted in the feces without being absorbed. Although the concentration of enrofloxacin in feces alone does not prove that it is eliminated through the liver, it can be an important reference. More importantly, the presence of antibiotics in feces from farming represents an important route into human life, and concerns about antibiotics in feces seems to be more relevant to environmental protection [35]. We observed that the cumulative recovery of enrofloxacin in feces was 261.06 ± 49.22 mg and that of ciprofloxacin was 0.33 ± 0.07 mg, which accounted for (25.73 ± 10.34)% of the administered dose. Enrofloxacin and ciprofloxacin as a percentage of the dose were reported to be (20.97 ± 2.29)% in urine and (19.23 ± 2.38)% in feces after oral administration of a single dose of enrofloxacin at 5.0 mg·kg^−1^ BW to pigs [16]. The results for the feces in this report were similar to those in our study. A high percentage of enrofloxacin is excreted through feces after enteral administration, which warrants proper fecal disposal during treatment.

Due to the lack of studies on donkeys, we collected the published MICs of several pathogenic bacteria isolated from horses against enrofloxacin and ciprofloxacin and present them in Table 4. Based on the previously published MICs for enrofloxacin on pathogens from equines, it was predicted to be effective for *E. coli* and *Salmonella* spp., *Staphylococcus* spp., *T. equigenitalis*, *Klebsiella* spp., *Proteus* spp., and *A. equuli*. The inhibitory effect of enrofloxacin on bacteria such as *Strept. Zooepidemicus*, *P. aeruginosa*, *R. equui*, *Strept. Equi*, and *Strept. equisimilis* seemed to not be significant. However, these MIC data are from a 1993 report, which is more than 30 years old, and the changes in pathogenic bacteria since then are not known, and therefore updated MIC data are needed to support the conclusions of this study. In human medical studies, an AUC_24_/MIC ≥ 100 or a C_max_/MIC ≥ 10 is considered to achieve the best therapeutic effect for treating bacterial infections [36,37]. However, it has been reported that in animals with normal immune systems, an AUC_24_/MIC of 40 may be sufficient to treat some infections [38]. So, based on these reports, enrofloxacin may not be an appropriate choice for donkeys when dealing with bacteria with an MIC > 0.25 mg·L^−1^. Whether it is possible to perform pharmacodynamic analysis on ciprofloxacin as a metabolite of enrofloxacin simply using C_max_ or AUC has not been concluded so far, and this requires follow-up studies.

Plasma protein binding affects the effect of drugs. Plasma protein binding of enrofloxacin or ciprofloxacin has not been reported in donkeys or horses. In dogs, the plasma protein binding of enrofloxacin was 34.74 ± 2.33% [39]. The plasma protein binding of enrofloxacin in steers and cows was 60.8 ± 1.05% and 59.4 ± 1.48% [40]. In calves, the plasma protein binding of enrofloxacin was 9.54–12.18% [41]. The recommended AUC_24_/MIC ratios in the present study are based on free plasma concentrations. However, for enrofloxacin and ciprofloxacin with moderate protein binding, the total plasma AUC was similar to the tissue AUC [15,39,42]. Fluoroquinolones are also moderately protein-bound in equine plasma and penetrate most equine tissues well [21]. The synovial fluid concentrations of enrofloxacin and ciprofloxacin were similar to horse plasma, and the interstitial fluid concentrations were similar to calf and pig plasma concentrations [15,21,42]. This evidence suggests that the total plasma AUC24/MIC ratio can be used to estimate the local interstitial fluid concentration at the site of infection [34,39].

Because of the high concentrations of enrofloxacin and ciprofloxacin in the urine in our study, enrofloxacin was predicted to be an effective option for dealing with donkey urinary tract infections. For example, *E. coli* (MIC_90_ = 0.03 mg·L^−1^ for enrofloxacin) is the main bacterium that causes urinary tract infections [43]. This may be effective against bacteria causing urinary tract infections such as *E. coli*, *Staphylococcus* spp., and *Klebsiella* spp., but other drugs should be chosen for *P. aeruginosa*. Ciprofloxacin is often used in human medicine to treat urinary tract infections [44]. As a metabolite of enrofloxacin, ciprofloxacin will have a positive impact on the effectiveness of enrofloxacin. However, due to the lack of results on the MICs of donkey-derived pathogens, care should be taken when determining the effective dose based on the MICs of equine-derived pathogens. There is a lack of information about the sensitivity of bacteria to the combination of enrofloxacin and ciprofloxacin, so this also would be a limitation of this study.

## 5. Conclusions

In the present study, it was obtained that intragastric administration of 7.5 mg·kg^−1^ BW of enrofloxacin was rapidly absorbed, slowly eliminated, and widely distributed by donkeys, and that this dose was predicted to be effective in donkeys against pathogenic bacteria with MICs < 0.25 mg·L^−1^.

## Figures and Tables

**Table 1:** Pharmacokinetic parameters of enrofloxacin and its metabolite ciprofloxacin in plasma of donkeys (single intragastric administration of 7.5 mg·kg^−1^ BW, *n* = 5).

Items	Enrofloxacin	Ciprofloxacin
T_1/2_ (h)	11.40 ± 6.40	7.25 ± 4.93
T_max_ (h)	0.55 ± 0.12	0.52 ± 0.08
C_max_ (mg·L^−1^)	2.46 ± 0.14	0.14 ± 0.03
AUC_0–∞_ (mg·L^−1^·h)	10.30 ± 3.37	0.24 ± 0.16
MRT (h)	7.88 ± 1.26	12.70 ± 12.63
Cl/F (L·kg^−1^·h^−1^)	0.81 ± 0.28	-
Vz/F (L·kg^−1^)	12.88 ± 6.70	-

T_1/2_, elimination half-life; AUC_0–∞_, the area under the curve; T_max_, time of maximum observed concentration; C_max_, maximum observed concentration; MRT, mean residence time; Cl/F, clearance; Vz/F, volume of distribution.

**Table 2:** Pharmacokinetic parameters of enrofloxacin and its metabolite ciprofloxacin in the urine of donkeys (single intragastric administration of 7.5 mg·kg^−1^ BW, *n* = 5).

Items	Enrofloxacin	Ciprofloxacin
T_1/2_ (h)	40.58 ± 14.59	15.71 ± 4.06
C_max_ (mg·L^−1^)	38.18 ± 8.56	15.94 ± 4.15
Time of maximum rate (h)	19.75 ± 1.30	16.00 ± 3.00
Maximum excretion rate (mg·h^−1^)	4.36 ± 1.10	1.82 ± 0.98
AURC_0–∞_ (mg)	47.00 ± 9.20	28.09 ± 10.23

T_1/2_, elimination half-life; C_max_, maximum observed concentration; AURC_0–∞_, area under rate curve.

**Table 3:** PK/PD parameters of enrofloxacin and its metabolite ciprofloxacin in plasma and urine of donkeys after a single intragastric administration (7.5 mg·kg^−1^ BW; *n* = 5).

Items		MIC Values (mg·L^−1^)						
		0.03	0.06	0.12	0.25	0.50	1.00	2.00
Plasma	AUC_24_ (mg·L^−1^·h)	AUC_24_/MIC						
Enrofloxacin	9.54	**318.00**	**159.00**	**79.50**	38.16	19.08	9.54	4.77
Ciprofloxacin	0.12	4.00	2.00	1.00	0.48	0.24	0.12	0.06
Enro + Cipro	9.66	322.00	161.00	80.50	38.64	19.32	9.66	4.83

AUC_24_, area under the concentration–time curve of 0–24 h. MIC, minimum inhibitory concentration. Predictive thresholds for antimicrobial drug efficacy (AUC_24_/MIC ≥ 50) are labeled in bold when available.

**Table 4:** Published minimum inhibitory concentrations (MICs) of enrofloxacin and ciprofloxacin (mg·L^−1^) against equine-disease-associated pathogens.

Pathogen	Enrofloxacin		Ciprofloxacin	
	MIC_50_^†^	MIC_90_ (Range) ^‡^	MIC_50_	MIC_90_ (Range)
*Salmonella* spp.	0.03	0.03	0.008	0.008
*E. coli*	0.015	0.03	0.008	0.008
*Strept. zooepidemicus*	1	1	1	1
*Staphylococcus* spp.	0.06	0.12	0.25	0.5
*T. equigenitalis*	0.03	0.06	0.06	0.12
*Klebsiella* spp.	0.015	0.03	0.015	(0.004–0.015)
*Proteus* spp.	0.12	(0.06–0.12)	0.015	(0.008–0.03)
*P. aeruginosa*	0.5	(0.25–0.05)	0.12	(0.06–0.12)
*A. equuli*	0.008	(0.008–0.12)	0.004	(0.004–0.03)
*R. equui*	2	-	1	-
*Strept. equi*	2	-	1	-
*Strept. equisimilis*	1	(0.5–2)	0.5	(0.25–1)

MICs data were reported by Ensink et al., 1993 [20]. ^†^ MIC_50_, the concentration in μg·mL^−1^ at which 50% of isolates were inhibited in growth. ^‡^ MIC_90_, the concentration in μg·mL^−1^ at which 90% of isolates were inhibited in growth. When MIC_90_ has no data, the concentration range is shown in parentheses.

**Table 5:** Basic information about experimental animals.

Items	Donkey					$\bar{x}$ ± *sd*
	1	2	3	4	5	
Body weight (kg)	171.00	185.00	158.00	105.00	119.00	147.60 ± 34.20
Single oral dose (mg·kg^−1^)	7.50	7.50	7.50	7.50	7.50	-
Administered dose (mg)	1282.50	1387.5	1185.00	787.50	892.50	1107.00 ± 229.63
Concentrated feed intake (kg·d^−1^)	1.50	1.50	1.50	1.50	1.50	-
Coarse fodder intake (kg·d^−1^)	2.15	1.96	1.87	1.73	1.69	1.88 ± 0.17
Total feces volume (kg)	31.65	31.65	39.73	31.80	29.80	32.93 ± 3.48
Water intake (L·d^−1^)	7.62	6.51	9.29	8.87	6.25	7.71 ± 1.22
Total urine volume (L)	11.84	10.16	14.21	12.86	11.20	12.05 ± 1.39

## Data Availability

The data not presented in the manuscript are available for consultation after a reasonable request to the corresponding author.
